# Supplementary material for: CXCL10 is a Tumor Microenvironment and Immune Infiltration Related Prognostic Biomarker in Pancreatic Adenocarcinoma
Source: Front Mol Biosci. 2021 Feb 18;8:611508. doi: 10.3389/fmolb.2021.611508 (PMC7930611; doi:10.3389/fmolb.2021.611508)
Supplement: Supplementary file 1 [file table1.docx]

**SUPPLEMENTARY MATERIAL**

**CXCL10 is a Tumor Microenvironment and Immune Infiltration Related Prognostic Biomarker in Pancreatic** **Adenocarcinoma**

Huimin Huang^1^, Wangxiao Zhou^2^, Renpin Chen^3^,Bingfeng Xiang^4^,Shipeng Zhou^1^, Linhua Lan^1^*

^1^Key Laboratory of Diagnosis and Treatment of Severe Hepato-Pancreatic Diseases of Zhejiang Province, The First Affiliated Hospital of Wenzhou Medical University, Wenzhou, China.

^2^State Key Laboratory for Diagnosis and Treatment of Infectious Diseases, The First Affiliated Hospital, College of Medicine, Zhejiang University, Hangzhou, China.

^3^Department of Gastroenterology, The First Affiliated Hospital of Wenzhou Medical University, Wenzhou, China.

^4^Department of Emergency Intensive Care Unit, The Cangnan Affiliated Hospital of Wenzhou Medical University, Wenzhou, China.

***Correspondence**

Linhua Lan

E-mail: [paullee90@wmu.edu.cn](mailto:paullee90@wmu.edu.cn)

**
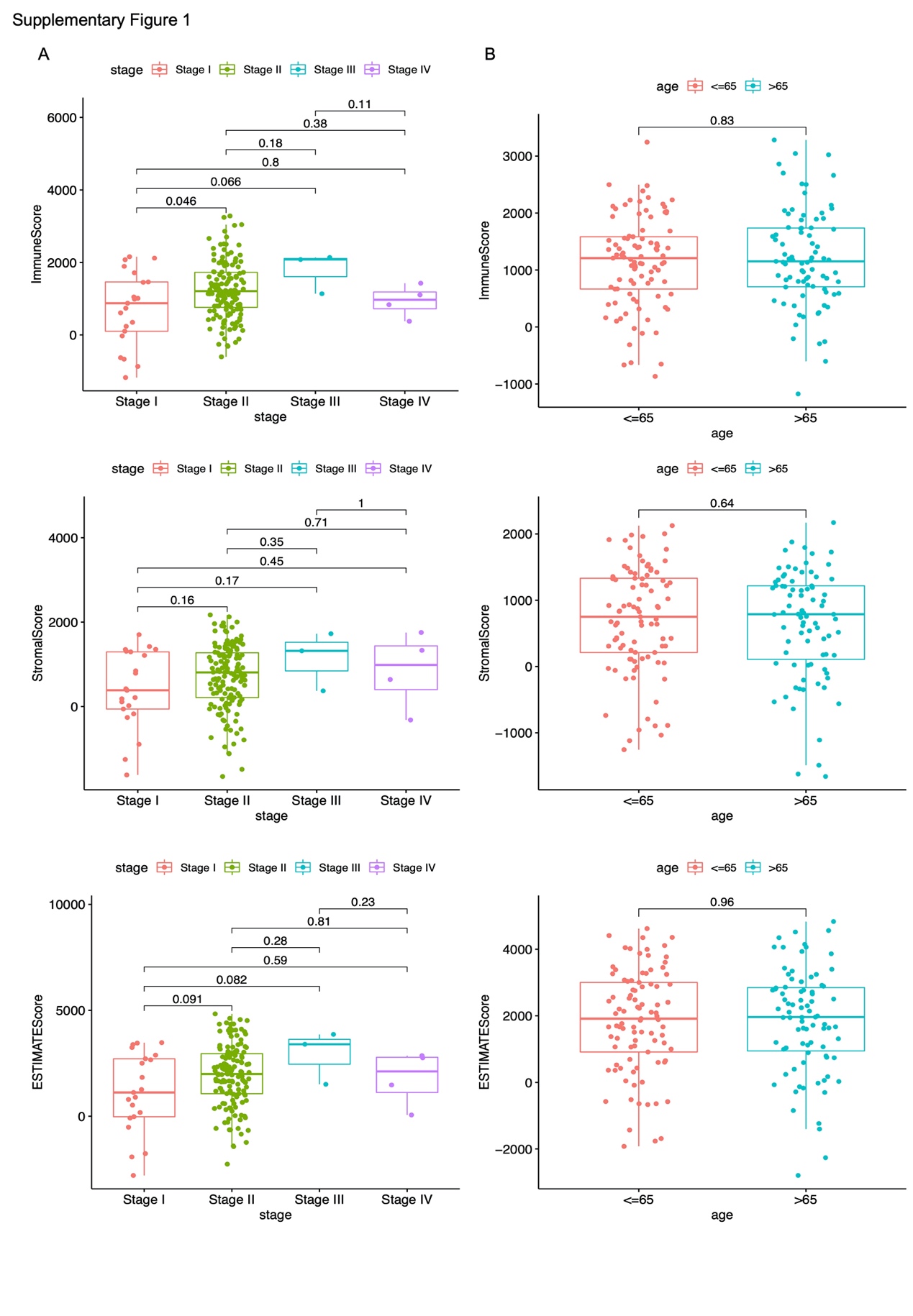
**

**Supplementary Figure 1.** Correlation between scores and pathology stage and age. **(A)** The immune scores were positively related to stage I and II disease (*p*=0.046). **(B)** No significance was found between the scores and age.


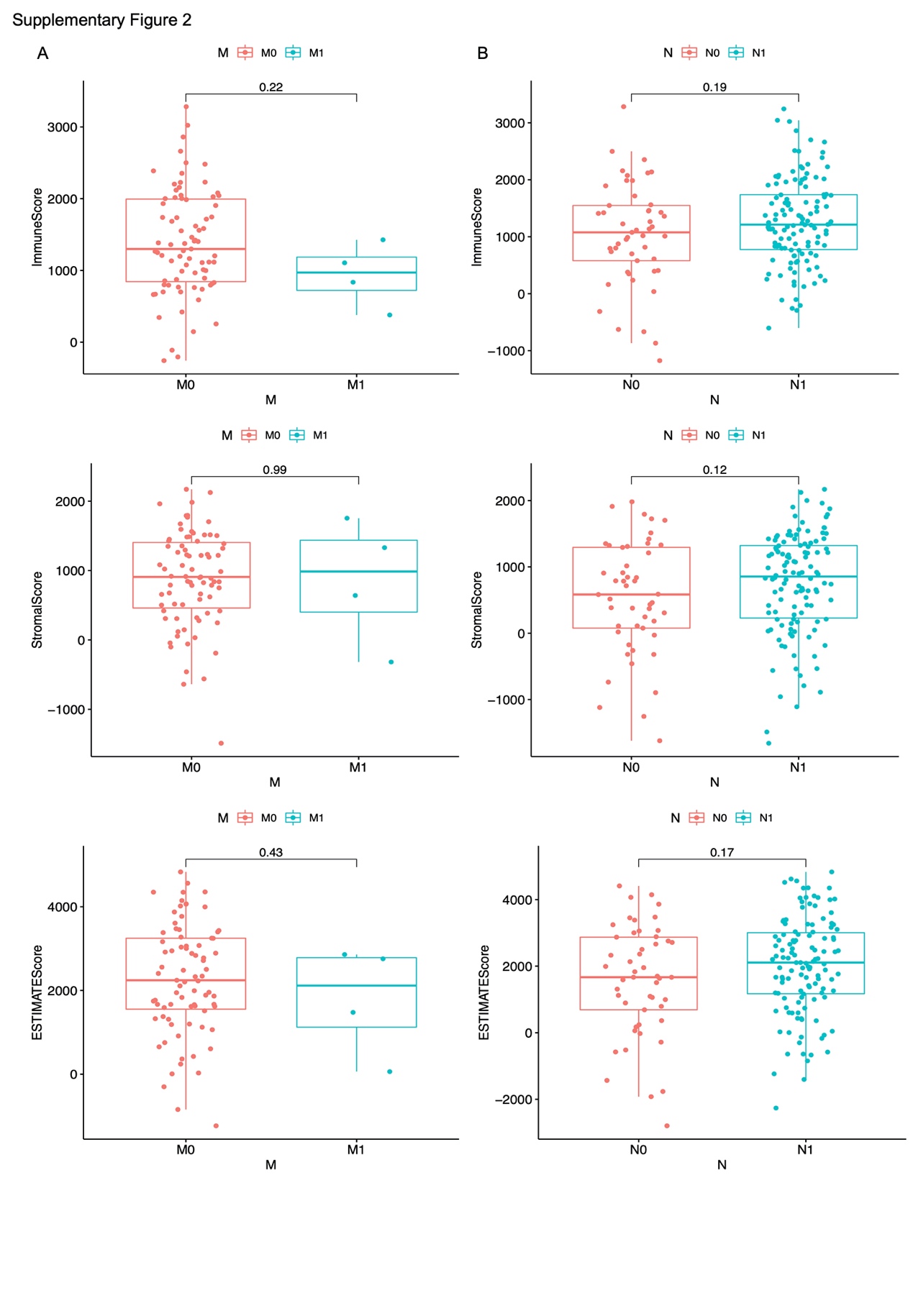


**Supplementary Figure 2.** Correlation between scores and M and N stage. **(A-B)** No significance was found between the scores and M and N stage.


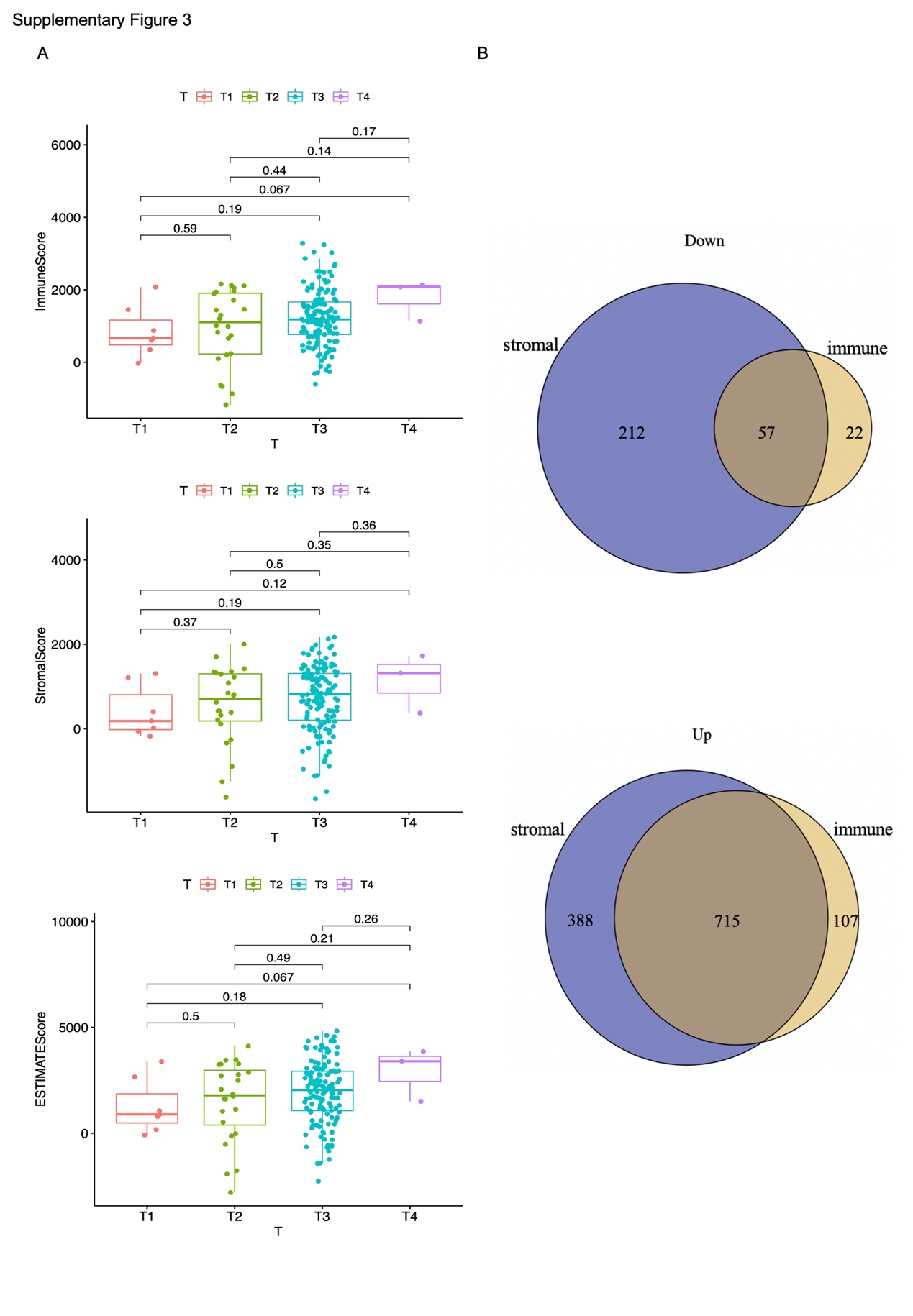


**Supplementary Figure 3.** Correlation between scores and T stage and Venn diagram analysis. **(A)** No significance was found between the scores and T stage. **(B)** Venn diagram analysis of up-regulated and down-regulated differentially expressed genes based on immune scores and stromal scores.


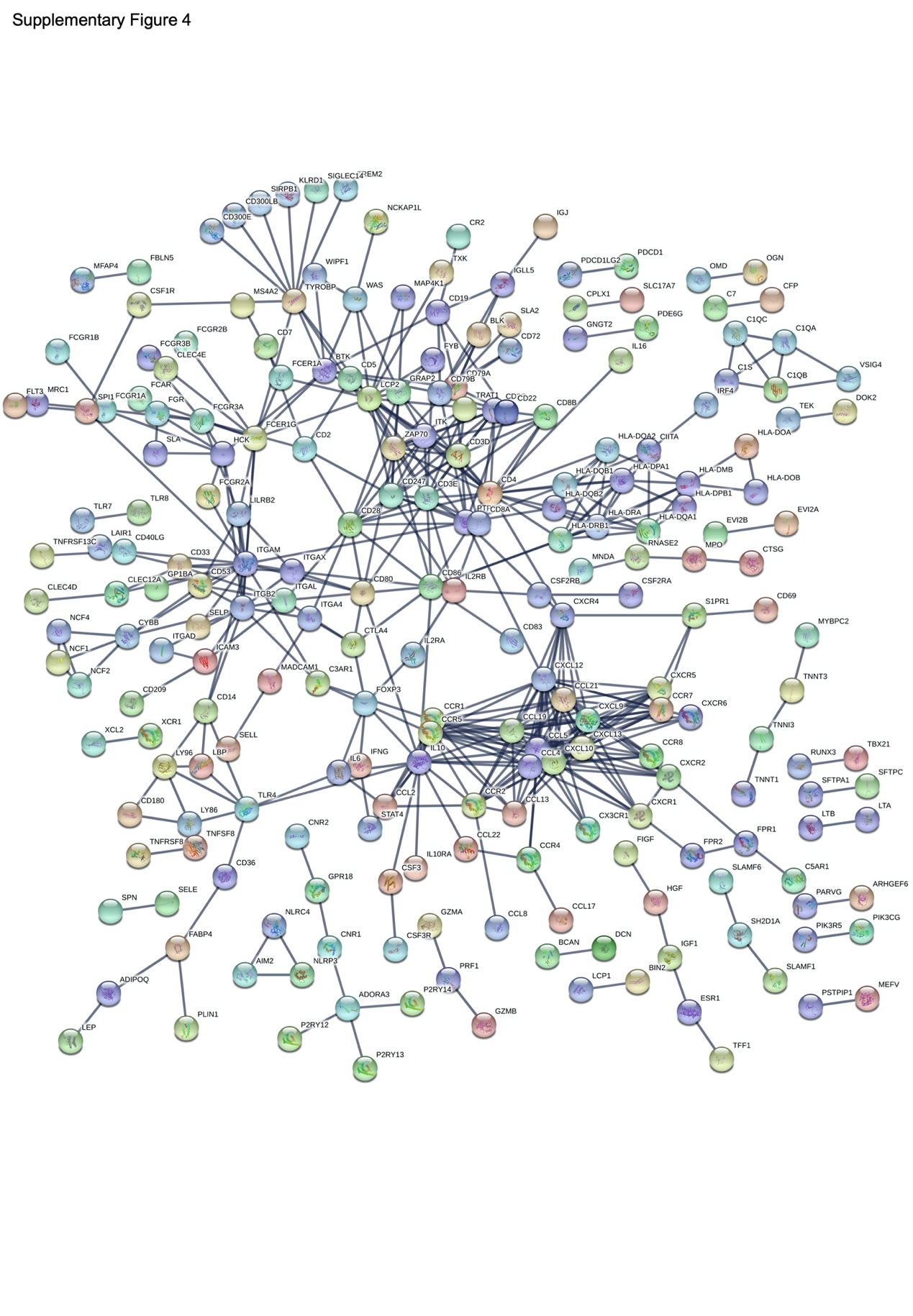


**Supplementary Figure 4.** PPI network construction. PPI network construction based on the STRING database covered 222 nodes and 476 edges with interaction confidence value more than 0.99.

**
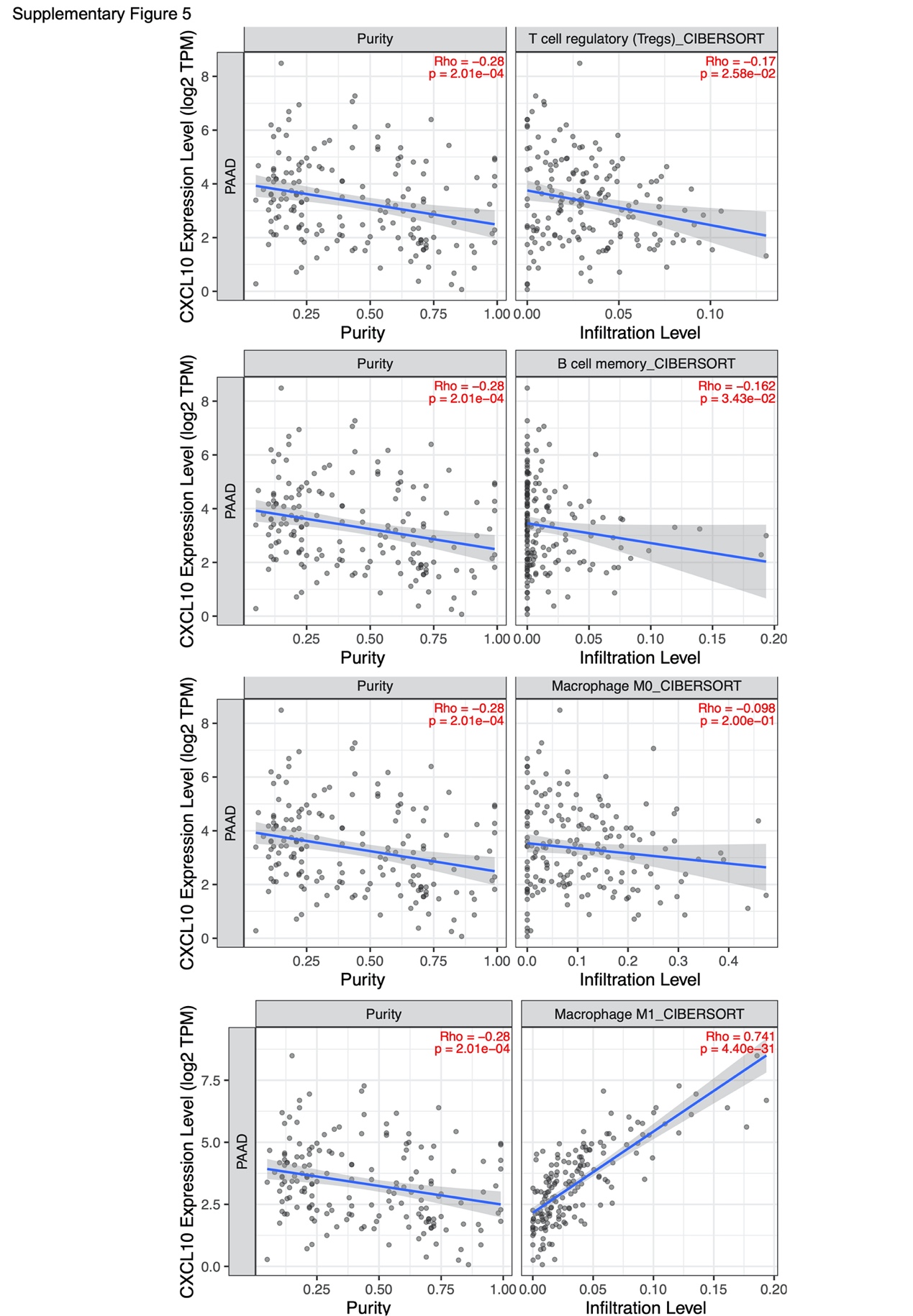
**

**Supplementary Figure 5.** Cumulative survival analysis. Cumulative survival analysis was performed to validate the correlation of CXCL10 and four immune cells.

Supplementary Table 1. Clinico-pathological characteristics statistics of PAAD patients from TCGA database

| Clinical characteristics |  | | Total | | % |
| --- | --- | --- | --- | --- | --- |
| Age at diagnosis (year)  Gender  Grade  Stage  T classification  M classification  N classification | <=65  >65  Female  Male  G1  G2  G3  G4  Ⅰ  Ⅱ  Ⅲ  Ⅳ  T1  T2  T3  T4  M0  M1  N0  N1 | 93  83  80  96  30  94  48  2  21  145  3  4  7  24  140  3  79  4  49  122 | | 52.8  47.2  45.5  54.5  17.2  54.0  27.6  1.1  12.1  83.8  1.7  2.3  4.0  14.0  80.5  1.7  95.2  4.8  28.7  71.3 | |

Supplementary Table 2. Enriched gene sets

| C7 collection | Gene set name | NES | | NOM p-val | |
| --- | --- | --- | --- | --- | --- |
| CXCL10 high expression    CXCL10 low expression | GSE13485_CTRL_VS_DAY3_YF17D_VACCINE_PBMC_DN  GSE14000_UNSTIM_VS_4H_LPS_DC_TRANSLATED_RNA_DN  GSE14415_INDUCED_TREG_VS_FOXP3_KO_INDUCED_TREG_IL2_CULTURE_UP  GSE15330_LYMPHOID_MULTIPOTENT_VS_MEGAKARYOCYTE_ERYTHROID_PROGENITOR_IKAROS_KO_DN  GSE19888_ADENOSINE_A3R_INH_VS_ACT_WITH_INHIBITOR_PRETREATMENT_IN_MAST_CELL_UP  GSE2706_UNSTIM_VS_8H_R848_DC_DN  GSE40685_TREG_VS_FOXP3_KO_TREG_PRECURSOR_DN  GSE43863_NAIVE_VS_MEMORY_TH1_CD4_TCELL_D150_LCMV_UP  GSE8835_CD4_VS_CD8_TCELL_CLL_PATIENT_UP  GSE18791_UNSTIM_VS_NEWCATSLE_VIRUS_DC_1H_UP  GSE21670_UNTREATED_VS_TGFB_TREATED_STAT3_KO_CD4_TCELL_UP  GSE34156_UNTREATED_VS_24H_NOD2_AND_TLR1_TLR2_LIGAND_TREATED_MONOCYTE_DN  GSE37533_UNTREATED_VS_PIOGLIZATONE_TREATED_CD4_TCELL_PPARG2_AND_FOXP3_TRASDUCED_UP | 2.41  2.34  2.39  2.36  2.41  2.31  2.31  2.31  2.35  -1.47  -1.39  -1.56  -1.53 | 0  0  0  0  0  0  0  0  0  0.034  0.037  0.022  0.020 | |  |

C7 collection: c7.all.v.7.1symbols.gmt from MSigDB. NES: normalized enrichment score. NOM: nominal *p-*value. Gene sets with NOM *p*-value less than 0.05 was considered as statistical significance.

Supplementary Table 3. The intersection of difference analysis and correlation analysis

| Tumor infiltrated immune cells | Correlation test（*p*-value） | | Difference test（*p*-value） |
| --- | --- | --- | --- |
| B-cells memory  T-cells regulatory  macrophages M0  macrophages M1 | 0.004  <0.001  <0.001  <0.001 | 0.032  <0.001  0.013  <0.001 | |
