# Supplementary material for: CXCL10 is a Tumor Microenvironment and Immune Infiltration Related Prognostic Biomarker in Pancreatic Adenocarcinoma
Source: Front Mol Biosci. 2021 Feb 18;8:611508. doi: 10.3389/fmolb.2021.611508 (PMC7930611; doi:10.3389/fmolb.2021.611508)
Supplement: Supplementary file 2 [file table2.docx]

**CXCL10 is a Tumor Microenvironment and Immune Infiltration Related Prognostic Biomarker in Pancreatic** **Adenocarcinoma**

Huimin Huang^1^, Wangxiao Zhou^2^, Renpin Chen^3^,Bingfeng Xiang^4^,Shipeng Zhou^1^, Linhua Lan^1^*

^1^Key Laboratory of Diagnosis and Treatment of Severe Hepato-Pancreatic Diseases of Zhejiang Province, The First Affiliated Hospital of Wenzhou Medical University, Wenzhou, China.

^2^State Key Laboratory for Diagnosis and Treatment of Infectious Diseases, The First Affiliated Hospital, College of Medicine, Zhejiang University, Hangzhou, China.

^3^Department of Gastroenterology, The First Affiliated Hospital of Wenzhou Medical University, Wenzhou, China.

^4^Department of Emergency Intensive Care Unit, The Cangnan Affiliated Hospital of Wenzhou Medical University, Wenzhou, China.

***Correspondence**

Linhua Lan

E-mail: [paullee90@wmu.edu.cn](mailto:paullee90@wmu.edu.cn)

Total words:

Figures: 10

Supplementary figures: 5

Supplementary tables: 3

**Keywords:** CXCL10, pancreatic adenocarcinoma, tumor microenvironment, tumor immune infiltration, prognosis

**ABSTRACT**

Pancreatic adenocarcinoma (PAAD) is the 10th most common cancer worldwide and the outcomes for patients with the disease remain extremely poor. Precision biomarkers are urgently needed to increase the efficiency of early diagnosis and to improve the prognosis of patients. The tumor microenvironment (TME) and tumor immune infiltration are thought to impact the occurrence, progression and prognosis of PAAD. Novel biomarkers excavated originating from the TME and immune infiltration may be effective in predicting the prognosis of PAAD patients. In the current study, the ESTIMATE and CIBERSORT algorithms were applied to estimate the division of immune and stromal components, and the proportion of tumor-infiltrating immune cells in 182 PAAD cases downloaded from The Cancer Genome Atlas database. Intersection analyses of the Protein-Protein Interaction networks and Cox regression analysis identified the chemokine (CXC-motif) ligand 10 (CXCL10) as a predictive biomarker. We verified that CXCL10 in the TME negatively correlates with prognosis in PAAD and positively correlates with tumor cell differentiation. GSE62452 from the GEO database and cumulative survival analysis were performed to validate CXCL10 expression as an independent prognostic indicator. We also found that memory B cells, regulatory T cells and macrophages M0 and M1 were correlated with the expression of CXCL10 indicating that expression of CXCL10 influenced the immune activity of the TME. Our data suggest that CXCL10 is beneficial as a prognostic indicator in PAAD patients and highlights the potential for immune taregeted therapy in the treatment of PAAD.

**INTRODUCTION**

Pancreatic adenocarcinoma (PAAD) is one of the most deadly malignant tumors and is ranked the seventh leading cause of cancer death (n = 432,000)(Bray et al., 2018). PAAD is commonly diagnosed in the advanced stage and currently no effective therapies are available(Yan et al., 2017). The prognosis in PAAD is highly unsatisfactory with 1-year survival less than 25% and 5-year survival is no more than 5%(Ottenhof et al., 2009). There is an urgent clinical need to identify novel biomarkers for the diagnosis and prognosis of PAAD.

Recently, research has focused on the tumor microenvironment (TME) and its role in cancer. The TME consists of the extracellular matrix, soluble molecules and tumor stromal cells(Thomas et al., 2019). Multiple studies have demonstrated that the TME can improve the invasiveness of tumor cells by modulating proliferation, chemotherapy resistance, immune escape and metastasis(Chen et al., 2015; J. H. Park et al., 2017). Immune and stromal cells are the two main types of non-tumor components in TME which have been considered to be valuable for tumor diagnosis and prognosis evaluation(Wormann et al., 2014; Xu et al., 2014). T cells, B cells and macrophages are chemotactic constituents within the TME. Several studies have demonstrated the role of stromal cells in extracellular matrix remodeling and tumor angiogenesis. Also, PAAD is characterized by an intense stromal desmoplastic reaction around the cancer cells(Bussard et al., 2016; Ligorio et al., 2019). Previous studies have demonstrated that infiltrating immune cells can be isolated from tumors suggesting that tumor immune infiltration is a crucial biological processes occur as a result of immune cell migration from the blood into the TME(Ge et al., 2019; R. Liu et al., 2020). Multiple reports have suggested that the proportion and functional alterations of different tumor infiltrated immune cells contribute to the initiation and progression of PAAD(Meng et al., 2020; Tian et al., 2020). However, the regulatory mechanisms of immune cells, stromal components of TME and tumor immune infiltration in PAAD remain largely unknown.

Based on the essential roles of the immune and stromal components within the TME, and tumor immune infiltration in carcinogenesis and progression, molecules extracted from the TME and tumor immune infiltration cells could have major potential as biomarkers in PAAD. Considering the pivotal roles of immune, stromal cells and tumor immune infiltration in PAAD, we first calculated immune and stromal scores and quantified the proportion of tumor immune cells using the Estimation of Stromal and Immune cells in Malignant Tumors using Expression data (ESTIMATE) algorithm. We used the CIBERSORT computational method to identify novel and potential therapeutic targets in PAAD. From differential gene expression analysis generated by comparing the immune and stromal components, we identified chemokine (CXC-motif) ligand 10 (CXCL10) as a potential predictive biomarker. CXCL10 belongs to the subfamily of the CXC chemokines identified as the main chemokine family in humans(Wu et al., 2020). Originally, CXCL10 is shown to be pro-inflammatory, proliferative and is associated with advanced human cancer(Dai et al., 2020; Lunardi et al., 2015). In this study, using bioinformatics analyses, we demonstrated that CXCL10 was a potentially valuable biomarker for alterations within the TME in PAAD.

**MATERIALS AND METHODS**

***Gene Expression Data***

A total of 182 cases of PAAD transcriptome FPKM data and clinico-pathological characteristics were downloaded from The Cancer Genome Atlas (TCGA) database ([https://portal.gdc.cancer.gov](https://portal.gdc.cancer.gov/)). These cases included 4 normal cases and 178 tumor cases. Due to the small scale of the normal samples in TCGA database, we used the UCSC Xena database (<https://xenabrowser.net/datapages/>) to obtain gene expression RNAseq FPKM data from the GTEx cohort. Gene expression data from these two databases were combined with the ‘normalize Between Arrays’ function in R language (version 4.0.2). To verify the precision of gene expression, mRNA expression data in PAAD were searched and downloaded from the GEO DataSets in NCBI (<https://www.ncbi.nlm.nih.gov/gds/>) using the keywords “pancreatic cancer”, “PAAD”, “microarray” and “adjacent”. The GSE62452 cohort contained 69 tumor samples and 61 adjacent normal tissue samples that were selected for further analysis.

***ESTIMATE Algorithms***

The ESTIMATE algorithms included three kinds of scores, specifically an immune score, a stromal score and an ESTIMATE score. The immune and stromal score were calculated based on the relative proportion of the immune and stromal elements. ESTIMATE scores were the sum of the immune and stromal scores. Three scores were calculated from the features of “limma” and “estimate” packages in R language loaded from gene expression files.

***Differential Expression Analysis and Enrichment of Genes***

Based on the results of ESTIMATE algorithms, all PAAD cases were divided into high and low groups according to the immune and stromal scores, respectively. The package named “pheatmap” was run to screen the heat-maps of the immune and stromal scores. The “limma” package from R language was performed to analyze the differentially expressed genes in the immune and stromal score groups with a threshold value of *p*<0.05 and |log fold change|>1 by a Wilcoxon rank-sum test.

***GO and KEGG Enrichment Analyses***

Gene Ontology (GO) and Kyoto Encyclopedia of Genes and Genomes (KEGG) pathway enrichment analysis of the 772 differentially expressed genes were selected by the “clusterProfiler”, “ggplot2”, “org.Hs.eg.db” and “enrichplot” packages. Both the *p*-values and *q-*values less than 0.05 of the samples were considered to be significantly enriched.

***PPI Network Construction and Cox Regression Analysis***

The Search Tool for the Retrieval of Interacting Gene (STRING) database was used to establish the Protein-Protein Interaction (PPI) network. Nodes where the interactive relationships were greater than 0.99 were selected to build the network. R language with the aid of package “survival” was set up for univariate Cox regression analysis which listed the top 16 genes ordered by the *p* values (*p*<0.05).

***Analysis of Gene Expression, Survival and Clinico-pathological Parameters***

Gene expression and survival analysis were performed using R language combined the packages “limma”, “beeswarm” and “survival”. Based on the Kruskal-Wallis rank-sum test, the “ggpubr” package in R language was run to explore the relationships between the stromal and immune scores, and the clinico-pathological parameters. Multivariate independent prognostic analysis was employed with the “survival” package in R language. A difference of *p* < 0.05 indicated statistical significance.

***Gene Set Enrichment Analysis***

C7.all.v.7.1symbols.gmt from MSigDB was used as the target set to carry out Gene Set Enrichment Analysis (GSEA) analysis of all tumor cases with the software GSEA (version 4.1.0) downloaded from the Broad Institute. Only gene sets with NOM *p*-value <0.05 were considered as statistically significant.

***Evaluation of Tumor Infiltrated Immune Cells***

The fractions of the tumor infiltrated immune cells of all tumor cases were calculated using the CIBERSORT algorithm. When the *p-*value of CIBERSORT was less than 0.05, the data were filtered and selected for further analysis with the “limma” package in R language. Difference and correlation analyses were applied using the “limma”, “vioplot”, “gglot2”, “ggpubr” and “ggExtra” packages in R language. *P-*values less than 0.05 were regarded as statistically significant using the Wilcoxon rank-sum and Pearson coefficient tests. Also, cumulative survival analysis was based on the Tumor Immune Estimation Resource (Timer) 2.0 database (<http://timer.cistrome.org/>).

**RESULTS**

**Flowchart of the Data Analysis Procedure**

A flowchart of the data analysis procedure used in this study is summarized in **Figure 1.** The fractions of tumor infiltrated immune cells and proportions of the immune and stromal component in the 182 cases of PAAD downloaded from TCGA were evaluated using the ESTIMATE and CIBERSORT algorithms. The tumor samples were evaluated based on the immune, stromal and ESTIMATE scores, and each cohort included its own set of differentially expressed genes. A total of 772 overlapping differentially expressed genes were found in the groups from the up & down-regulated immune and stromal scores. Using PPI network construction and Cox regression analysis to calculate the differentially expressed genes, an intersection analysis was applied to the datasets. Based on these analyses, we found that CXCL10 was closely associated with the prognosis of PAAD patients. Subsequently, analysis of survival, clinical correlation, validation and correlation were performed to evaluate the prognostic value of CXCL10.

**Remodeling of Tumor Immune Infiltration and TME Indices are Closely Related to Clinico-pathological Characteristics of PAAD**

A previous study has been reported that the ESTIMATE algorithms, either the stromal or the immune score, are indicators of patient survival, relapse, metastasis and chemotherapeutic drug resistance . Higher immune or stromal scores represented larger amounts of immune or stromal components in the TME. The feasible association of the scores and clinico-pathological characteristics were then explored. The clinico-pathological characteristics of the PAAD patients are summarized in **Supplementary Table1**, which were analyzed after detecting the data. The data showed the immune, stromal and ESTIMATE scores were all significantly associated with gender as well as G1 and G2 histologically graded disease (**Figure 2**). The *p* values of gender were 0.039, 0.037 and 0.034 respectively, whilst the *p* values between G1 and G2 of histological grades were 0.035, 0.02 and 0.038, respectively. The immune scores were also positively related to stage I and II disease (**Supplementary Figure 1A**, *p*=0.046), however, no significance was found between the scores and the indicated clinico-pathological features (**Supplementary Figure 1B&2&3A**). These data suggested that the immune and stromal scores were closely related to the progression of PAAD.

**Identification of Differentially Expressed Genes in Tumor Infiltrating Immune Cells and the TME**

According to the median score of the stromal and immune scores, the PAAD patients were divided into two groups. Differential expression analysis identified differentially expressed genes in the immune (both low and high scores) and stromal score groups (both low and high scores). Heat-maps displayed the differential expression of these genes. The up-regulated (logFC>0) genes represent genes that were significantly elevated compared to the down-regulated genes (logFC<0). Based on the immune scores, a total of 901 differentially expressed genes were acquired amongst which 822 genes were up-regulated and 79 genes were down-regulated **(Figure 3A**). Similarly, 1372 differentially expressed genes were obtained in the two stromal score groups amongst which 1103 genes were up-regulated and 169 genes were down-regulated (**Figure 3B**). Also, intersections between the high stromal and immune score group or the low stromal and immune score group were visualized in a Venn diagram. Data showed 715 common differentially expressed genes that were up-regulated in both the immune and stromal score groups, and 57 common differentially expressed genes that were down-regulated in both the immune and stromal score groups (**Supplementary Figure 3B**). A total of 772 differentially expressed genes may be crucial components of the TME.

**Differentially Expressed Genes Have Principal Roles in Mediating Immune-related Functions**

GO and KEGG pathway enrichment analyses were conducted to identify the latent molecular mechanisms of 772 differentially expressed genes. The results of the GO functional enrichment analysis indicated that the differentially expressed genes were enriched in immune-related functions including T-cell activation, regulation of lymphocyte activation and leukocyte migration (**Figure 4A**). The results of the KEGG functional enrichment analysis showed differentially expressed genes mainly enriched in cytokine-cytokine receptor interactions, hematopoietic cell lineages and chemokine signaling pathways (**Figure 4B**). These data highlighted both functional enrichment analyses appeared to focus on immune-related functions suggesting that the participation of immune components have principal roles in the TME in PAAD.

**CXCL10 is the Only Intersectional Gene in the PPI Network Construction and Univariate Cox Regression Analysis**

To visualize potential interactions between the differentially expressed genes, PPI network construction based on the STRING database was performed that covered 222 nodes and 476 edges (**Supplementary Figure 4**). The top 30 differentially expressed genes were sorted and summarized by the number of nodes (**Figure 5A**). Univariate Cox regression analysis was performed to further investigate the significant elements of these differentially expressed genes in the survival of PAAD and the top 16 genes sorted by *p-*value were identified (**Figure 5B**). According to the intersection analysis of the PPI network and the univariate Cox regression analysis, CXCL10 was shown to be the only overlapping element across these two analyses (**Figure 5C**).

**CXCL10 is Strongly Correlated with Prognosis and the Clinico-pathological Characteristics of PAAD Patients**

A previous study demonstrated that CXCL10 is a pro-inflammatory chemokine and chemo-attractant for T cells. Overexpression of CXCL10 has been shown to promote tumor growth, migration and invasion via targeting of the cognate receptor chemokine (CXC-motif) receptor (CXCR3)(Lunardi et al., 2015). In this study, based on the median expression of CXCL10, all PAAD cases were divided into high or low CXCL10 expression groups. The Wilcoxon rank-sum test was carried out and showed the correlation of CXCL10 expression with clinical characteristics. No significant difference in the expression of CXCL10 was found between normal and tumor cases from the TCGA database (**Figure 6A**). Interestingly, when the normal cases were added from the GTEx database, the expression of CXCL10 in the tumor cases was higher compared to the normal cases **(Figure 6B**, *p*<0.001). Survival analysis indicated that CXCL10 expression was negatively related to outcomes in PAAD patients (**Figure 6C**). Also, the expression of CXCL10 correlated with gender, G1, G2 and G3 of histologic grades with *p* values of 0.015, 0.029 and 0.034, respectively (**Figure 7A-B**). All of the above results show that over-expression of CXCL10 in the TME had a negative relationship with prognosis in PAAD and was positively related to tumor cell differentiation. No significance was found in the indicated clinical parameters and CXCL10 expression (**Figure 7C-G**).

**Validation of CXCL10 Expression and Survival in PAAD**

GSE62452 from the GEO databases was analyzed to verify the expression of CXCL10 in PAAD. The expression of CXCL10 was higher in the tumor samples compared to the normal cases (**Figure 6D** , *p*<0.001). Higher CXCL10 expression was associated with poorer survival compared to low CXCL10 expression (**Figure 6E**, *p*=0.016). Moreover, the multivariate independent prognostic analysis revealed that the expression of CXCL10 was an independent prognostic indicator for PAAD (*p*=0.035) **(Figure 6F**). Based on these data, we suggest that CXCL10 may be a potential biomarker that reflects the status of TME in PAAD.

**CXCL10 Impacts the Immune Activity of the TME**

To further confirm the association of CXCL10 expression and the immune microenvironment, C7 collection from MSigDB in GSEA was used to analyze data between the high and low CXCL10 expression groups. Most immune functional gene sets were enriched in the high CXCL10 expression group whilst few gene sets were enriched in the low CXCL10 expression group (**Figure 8, Supplementary Table 2**). The fraction of tumor infiltrated immune cells were analyzed using CIBERSORT and 22 kinds of immune cells in PAAD were constructed (**Figure 9**). Through the intersection of difference and correlation analyses, 3 kinds of tumor-infiltrating immune cells were negatively associated with the expression of CXCL10 which included memory B cells, regulatory T cells (Tregs) and macrophages M0. Macrophages M1 were shown to be positively correlated with CXCL10 expression (**Figure 10, Supplementary Table 3**).

To validate the correlation of CXCL10 expression in immune cells, cumulative survival analysis was performed. Indeed, the infiltration level of Tregs, B cell and macrophage M0-CIBERSORT subtype were negatively correlated with CXCL10 expression, while the infiltration level of macrophage M1-CIBERSORT subtype showed positively associated with CXCL10 expression (**Supplementary Figure 5**). Collectively, all the data prove that aberrant expression of CXCL10 impacts the immune activity of the TME which is a promising biomarker for the prognosis of PAAD patients.

**DISCUSSION**

Recently, remodeling of the TME has been shown to play a crucial role in the initiation and progression of multiple cancer types(Cheng et al., 2019; Jiang et al., 2020; S. A. Park et al., 2017). Also, several studies have been performed to dissect the features of the TME in PAAD(Hessmann et al., 2020; Karamitopoulou, 2019; Meng et al., 2020). As surgical resection is the only option for a limited number of eligible patients(Petrusel et al., 2020), specific signaling factors and molecules from TME involved in the carcinogenesis and progression of PAAD remain to be fully determined. Exploring the potential mechanisms of immune and stromal cells within the TME(Tsai et al., 2018) may be a promising strategy that could potentially inform the development of novel therapeutic options in PAAD. To date, the ESTIMATE algorithms could be applied to calculate the components of immune and stromal cells(H. Wang et al., 2019) to obtain corresponding immune and stromal scores. Our data from transcriptomic analysis based on PAAD samples revealed the proportion of immune and stromal components in TME contributed to the prognosis of PAAD. These consequences highlighted the significance of exploring the association of tumor cells and immune cells which provided constructive insight for developing much more effective treatment regimen. Though immunotherapy has become support of cancer treatment and brings new hope for PAAD(Fritz et al., 2019), the developments of individual patient confront with tough challenges due to side effects(Sahin et al., 2017). It is necessary to investigate some novel candidates for the immunotherapy of PAAD. Here, we set out from transcriptomic analysis of PAAD, which implied the increased expression of CXCL10 was significantly associated with poor prognosis and advanced clinico-pathological characteristics.

CXCL10, known as interferon (IFN)-γ-induced protein 10 (IP-10), belongs to the CXC chemokine subfamily and contains a single and variable amino acid between two of the four highly conserved cysteine residues(Antonelli et al., 2014; Muller et al., 2010; Qian et al., 2019). Several studies have demonstrated the significant role of CXCL10 in tumor, including PAAD. The effects of CXCL10 are mediated by binding to CXCR3 receptor. Significantly higher levels of CXCL10 and CXCR3 are observed in PAAD specimens compared to those from chronic pancreatitis(Moin et al., 2018; Singh et al., 2007). Rencent studies have confirmed a stromal origin of CXCL10 in human tumors which is overexpressed in human pancreatic cancer and associate with poor survival of PAAD patients(Delitto et al., 2015). Recently, CXCL10 and CCL21 are demonstrated to promote migration of pancreatic cancer cells towards sensory neurons and increase the frequency of cancer-associated pain(Hirth et al., 2020). Moreover, High expression of CXCL10 is also contributed to chemotherapeutic gemcitabine(Delitto et al., 2015). Consistently, in our study, we also validated the bioinformatic characteristics of CXCL10, whereby CXCL10 expression was an independent predictor of poor survival in PAAD. Further work needs to investigate molecular mechanism of CXCL10 in regulating malignant phenotypes of PAAD.

Tumor infiltrated immune cells can regulate the proportion and distribution of immune cells in tumors and tumor inflammation is abnormally active especially in the early stage of carcinogenesis(Neviani et al., 2019). We found CXCL10 was correlated with early stage of PAAD which indicated potential role of CXCL10 in PAAD carcinogenesis. Recently, macrophage M0 is demonstrated harbor anti-tumorigenic activities to suppress PAAD cell growth by TNF-α secretion, but not M1 or M2(Tekin et al., 2020). This could be an explanation why CXCL10 is negatively related to macrophage M0 in our study. Another issue is that we found CXCL10 was positively correlated with macrophage M1. Macrophages M1, act as anti-tumoral immune components, are clarified to prevent immune escape in PAAD(Young et al., 2020). Tumor expressed galectin 9 activates dectin 1 on macrophage which subsequently promotes PAAD and enhances immune tolerance(Daley et al., 2017). Combined with the pro-inflammatory properties of macrophage M1, we speculated that aberrant up-regulation of CXCL10 and increased macrophage M1 may be an early event in the process of carcinogenesis.

Tumor-infiltrating Treg cells can suppress tumor-associated DC immunogenicity to promote immune tolerance in pancreatic cancer(Jang et al., 2017). Moreover, FOXP3^+^ Tregs recruited in TME contribute to immunosuppressive microenvironment which subsequently promote immune escape and metastasis of PAAD(X. Wang et al., 2017). Also, circulating Tregs are identified as prognostic factors and can predict chemotherapeutic response in unresectable PDAC patients(C. Liu et al., 2017). These works collectively emphasize infiltration of Tregs in TME of PAAD. However, in current work, we found CXCL10 was negatively correlated with Tregs. Previous study identified systemic dysfunction and plasticity of the immune macroenvironment, and found CD103^+^ Tregs were recruited at day 7 but decreased with tumor progression, indicating dynamic distribution of Tregs in TME during the process of tumor initiation and progression(Allen et al., 2020). Additionally, pancreatic stellate cells (PSCs) secrete CXCL10 induced by pancreatic cells to recruit CXCR3^+^ and FOXP3^+^ Tregs, which subsequently suppress adaptive immune responses and promote immunosuppression(Lunardi et al., 2015). Thus, we speculate that during the progression of PAAD, the correlation between the expression of CXCL10 in tumor cells and the abundance of Tregs cells will be weakened. Further work needs to focus on exploring the correlation between CXCL10 and different subtypes of Tregs, and then systematically understand the dynamic changes of CXCL10 in the progress of PAAD.

Collectively, based on the ESTIMATE algorithms, functional enrichment analysis, PPI network construction and Cox regression analysis, we suggest CXCL10 is a promising diagnostic and prognostic indicator in PAAD patients which has potential to provide novel immunotherapy insights for PAAD. Further studies are required to explore CXCL10 as a therapeutic target in the treatment of PAAD.

**ACKNOWLEDGEMENTS**

We gratefully acknowledge contributions from the TCGA, UCSC and GEO databases research Network. Thanks for all the members’ help in the visualization of graphical abstract.

**DATA AVAILABILITY STATEMENT**

Publicly available datasets were analyzed in this study. This data can be found here: The Cancer Genome Atlas database [(https://portal.gdc.cancer.gov)](file:///C:\Users\microsoft\Documents\研究课题\阿黄课题\第一篇2020-10-05\返修2020-11-28\(https:\portal.gdc.cancer.gov)), UCSC Xena database (http://xena. ucsc.edu/) and GEO DataSets in NCBI (<https://www.ncbi.nlm.nih.gov/gds/)>.

**AUTHOR CONTRIBUTIONS**

LL conceived and designed the study. HH and WZ collected and analyzed and interpreted the data. HH RC, BX and SZ participated in interpreting and revising the manuscript. LL and HH wrote the manuscript. All authors contributed to manuscript revision, read, and approved the submitted version.

**FUNDING**

This study was supported by the National Natural Science Foundation Youth Fund Project of China (No. 81902803) and Natural Science Foundation of Zhejiang Province (No.LY21H160057).

**SUPPLEMENTARY MATERIAL**

**Supplementary Figure 1.** Correlation between scores and pathology stage and age.

**Supplementary Figure 2.** Correlation between scores and M and N stage.

**Supplementary Figure 3.** Correlation between scores and T stage and Venn diagram analysis.

**Supplementary Figure 4.** PPI network construction.

**Supplementary Figure 5.** Cumulative survival analysis.

**Supplementary Table1.** Clinico-pathological characteristics statistics of PAAD patients from TCGA

**Supplementary Table2.** Enriched gene sets

**Supplementary Table3.** The intersection of difference analysis and correlation analysis

**Conflict of Interest:** The authors declare that the research was conducted in the absence of any commercial or financial relationships that could be construed as a potential conflict of interest.

**REFERENCES:**

Allen, B. M., Hiam, K. J., Burnett, C. E., Venida, A., DeBarge, R., Tenvooren, I., et al. (2020). Systemic dysfunction and plasticity of the immune macroenvironment in cancer models. *Nat Med. 26*(7), 1125-1134. doi:10.1038/s41591-020-0892-6

Antonelli, A., Ferrari, S. M., Giuggioli, D., Ferrannini, E., Ferri, C. and Fallahi, P. (2014). Chemokine (C-X-C motif) ligand (CXCL)10 in autoimmune diseases. *Autoimmun Rev. 13*(3), 272-280. doi:10.1016/j.autrev.2013.10.010

Bray, F., Ferlay, J., Soerjomataram, I., Siegel, R. L., Torre, L. A. and Jemal, A. (2018). Global cancer statistics 2018: GLOBOCAN estimates of incidence and mortality worldwide for 36 cancers in 185 countries. *CA Cancer J Clin. 68*(6), 394-424. doi:10.3322/caac.21492

Bussard, K. M., Mutkus, L., Stumpf, K., Gomez-Manzano, C. and Marini, F. C. (2016). Tumor-associated stromal cells as key contributors to the tumor microenvironment. *Breast Cancer Res. 18*(1), 84. doi:10.1186/s13058-016-0740-2

Chen, F., Zhuang, X., Lin, L., Yu, P., Wang, Y., Shi, Y., et al. (2015). New horizons in tumor microenvironment biology: challenges and opportunities. *BMC Med. 13*, 45. doi:10.1186/s12916-015-0278-7

Cheng, H. S., Lee, J. X. T., Wahli, W. and Tan, N. S. (2019). Exploiting vulnerabilities of cancer by targeting nuclear receptors of stromal cells in tumor microenvironment. *Mol Cancer. 18*(1), 51. doi:10.1186/s12943-019-0971-9

Dai, S., Liu, F., Qin, Z., Zhang, J., Chen, J., Ding, W. X., et al. (2020). Kupffer cells promote T-cell hepatitis by producing CXCL10 and limiting liver sinusoidal endothelial cell permeability. *Theranostics. 10*(16), 7163-7177. doi:10.7150/thno.44960

Daley, D., Mani, V. R., Mohan, N., Akkad, N., Ochi, A., Heindel, D. W., et al. (2017). Dectin 1 activation on macrophages by galectin 9 promotes pancreatic carcinoma and peritumoral immune tolerance. *Nat Med. 23*(5), 556-567. doi:10.1038/nm.4314

Delitto, D., Perez, C., Han, S., Gonzalo, D. H., Pham, K., Knowlton, A. E., et al. (2015). Downstream mediators of the intratumoral interferon response suppress antitumor immunity, induce gemcitabine resistance and associate with poor survival in human pancreatic cancer. *Cancer Immunol Immunother. 64*(12), 1553-1563. doi:10.1007/s00262-015-1760-y

Fritz, J. M. and Lenardo, M. J. (2019). Development of immune checkpoint therapy for cancer. *J Exp Med. 216*(6), 1244-1254. doi:10.1084/jem.20182395

Ge, P., Wang, W., Li, L., Zhang, G., Gao, Z., Tang, Z., et al. (2019). Profiles of immune cell infiltration and immune-related genes in the tumor microenvironment of colorectal cancer. *Biomed Pharmacother. 118*, 109228. doi:10.1016/j.biopha.2019.109228

Hessmann, E., Buchholz, S. M., Demir, I. E., Singh, S. K., Gress, T. M., Ellenrieder, V., et al. (2020). Microenvironmental Determinants of Pancreatic Cancer. *Physiol Rev. 100*(4), 1707-1751. doi:10.1152/physrev.00042.2019

Hirth, M., Gandla, J., Hoper, C., Gaida, M. M., Agarwal, N., Simonetti, M., et al. (2020). CXCL10 and CCL21 Promote Migration of Pancreatic Cancer Cells Toward Sensory Neurons and Neural Remodeling in Tumors in Mice, Associated With Pain in Patients. *Gastroenterology. 159*(2), 665-681 e613. doi:10.1053/j.gastro.2020.04.037

Jang, J. E., Hajdu, C. H., Liot, C., Miller, G., Dustin, M. L. and Bar-Sagi, D. (2017). Crosstalk between Regulatory T Cells and Tumor-Associated Dendritic Cells Negates Anti-tumor Immunity in Pancreatic Cancer. *Cell Rep. 20*(3), 558-571. doi:10.1016/j.celrep.2017.06.062

Jiang, Y., Wang, C. and Zhou, S. (2020). Targeting tumor microenvironment in ovarian cancer: Premise and promise. *Biochim Biophys Acta Rev Cancer. 1873*(2), 188361. doi:10.1016/j.bbcan.2020.188361

Karamitopoulou, E. (2019). Tumour microenvironment of pancreatic cancer: immune landscape is dictated by molecular and histopathological features. *Br J Cancer. 121*(1), 5-14. doi:10.1038/s41416-019-0479-5

Ligorio, M., Sil, S., Malagon-Lopez, J., Nieman, L. T., Misale, S., Di Pilato, M., et al. (2019). Stromal Microenvironment Shapes the Intratumoral Architecture of Pancreatic Cancer. *Cell. 178*(1), 160-175 e127. doi:10.1016/j.cell.2019.05.012

Liu, C., Cheng, H., Luo, G., Lu, Y., Jin, K., Guo, M., et al. (2017). Circulating regulatory T cell subsets predict overall survival of patients with unresectable pancreatic cancer. *Int J Oncol. 51*(2), 686-694. doi:10.3892/ijo.2017.4032

Liu, R., Hu, R., Zeng, Y., Zhang, W. and Zhou, H. H. (2020). Tumour immune cell infiltration and survival after platinum-based chemotherapy in high-grade serous ovarian cancer subtypes: A gene expression-based computational study. *EBioMedicine. 51*, 102602. doi:10.1016/j.ebiom.2019.102602

Liu, W., Ye, H., Liu, Y. F., Xu, C. Q., Zhong, Y. X., Tian, T., et al. (2018). Transcriptome-derived stromal and immune scores infer clinical outcomes of patients with cancer. *Oncol Lett. 15*(4), 4351-4357. doi:10.3892/ol.2018.7855

Lunardi, S., Lim, S. Y., Muschel, R. J. and Brunner, T. B. (2015). IP-10/CXCL10 attracts regulatory T cells: Implication for pancreatic cancer. *Oncoimmunology. 4*(9), e1027473. doi:10.1080/2162402X.2015.1027473

Meng, Z., Ren, D., Zhang, K., Zhao, J., Jin, X. and Wu, H. (2020). Using ESTIMATE algorithm to establish an 8-mRNA signature prognosis prediction system and identify immunocyte infiltration-related genes in Pancreatic adenocarcinoma. *Aging (Albany NY). 12*(6), 5048-5070. doi:10.18632/aging.102931

Moin, A. S. M., Cory, M., Choi, J., Ong, A., Dhawan, S., Dry, S. M., et al. (2018). Increased Chromogranin A-Positive Hormone-Negative Cells in Chronic Pancreatitis. *J Clin Endocrinol Metab. 103*(6), 2126-2135. doi:10.1210/jc.2017-01562

Muller, M., Carter, S., Hofer, M. J. and Campbell, I. L. (2010). Review: The chemokine receptor CXCR3 and its ligands CXCL9, CXCL10 and CXCL11 in neuroimmunity--a tale of conflict and conundrum. *Neuropathol Appl Neurobiol. 36*(5), 368-387. doi:10.1111/j.1365-2990.2010.01089.x

Neviani, P., Wise, P. M., Murtadha, M., Liu, C. W., Wu, C. H., Jong, A. Y., et al. (2019). Natural Killer-Derived Exosomal miR-186 Inhibits Neuroblastoma Growth and Immune Escape Mechanisms. *Cancer Res. 79*(6), 1151-1164. doi:10.1158/0008-5472.CAN-18-0779

Ottenhof, N. A., Milne, A. N., Morsink, F. H., Drillenburg, P., Ten Kate, F. J., Maitra, A., et al. (2009). Pancreatic intraepithelial neoplasia and pancreatic tumorigenesis: of mice and men. *Arch Pathol Lab Med. 133*(3), 375-381. doi:10.1043/1543-2165-133.3.375

Park, J. H., van Wyk, H., Roxburgh, C. S. D., Horgan, P. G., Edwards, J. and McMillan, D. C. (2017). Tumour invasiveness, the local and systemic environment and the basis of staging systems in colorectal cancer. *Br J Cancer. 116*(11), 1444-1450. doi:10.1038/bjc.2017.108

Park, S. A. and Surh, Y. J. (2017). Modulation of tumor microenvironment by chemopreventive natural products. *Ann N Y Acad Sci. 1401*(1), 65-74. doi:10.1111/nyas.13395

Petrusel, L., Bilibou, M., Drug, V., Leucuta, D. C., Seicean, R., Cainap, C., et al. (2020). Risk Factors in Pancreatic Adenocarcinoma: the Interrelation with Familial History and Predictive Role on Survival. *J Gastrointestin Liver Dis. 29*(3), 391-398. doi:10.15403/jgld-2529

Qian, L., Yu, S., Yin, C., Zhu, B., Chen, Z., Meng, Z., et al. (2019). Plasma IFN-gamma-inducible chemokines CXCL9 and CXCL10 correlate with survival and chemotherapeutic efficacy in advanced pancreatic ductal adenocarcinoma. *Pancreatology. 19*(2), 340-345. doi:10.1016/j.pan.2019.01.015

Sahin, I. H., Askan, G., Hu, Z. I. and O'Reilly, E. M. (2017). Immunotherapy in pancreatic ductal adenocarcinoma: an emerging entity? *Ann Oncol. 28*(12), 2950-2961. doi:10.1093/annonc/mdx503

Singh, L., Bakshi, D. K., Majumdar, S., Vasishta, R. K., Arora, S. K. and Wig, J. D. (2007). Expression of interferon-gamma- inducible protein-10 and its receptor CXCR3 in chronic pancreatitis. *Pancreatology. 7*(5-6), 479-490. doi:10.1159/000108965

Tekin, C., Aberson, H. L., Bijlsma, M. F. and Spek, C. A. (2020). Early macrophage infiltrates impair pancreatic cancer cell growth by TNF-alpha secretion. *BMC Cancer. 20*(1), 1183. doi:10.1186/s12885-020-07697-1

Thomas, D. and Radhakrishnan, P. (2019). Tumor-stromal crosstalk in pancreatic cancer and tissue fibrosis. *Mol Cancer. 18*(1), 14. doi:10.1186/s12943-018-0927-5

Tian, X., Xu, W., Wang, Y., Anwaier, A., Wang, H., Wan, F., et al. (2020). Identification of tumor-infiltrating immune cells and prognostic validation of tumor-infiltrating mast cells in adrenocortical carcinoma: results from bioinformatics and real-world data. *Oncoimmunology. 9*(1), 1784529. doi:10.1080/2162402X.2020.1784529

Tsai, S., McOlash, L., Palen, K., Johnson, B., Duris, C., Yang, Q., et al. (2018). Development of primary human pancreatic cancer organoids, matched stromal and immune cells and 3D tumor microenvironment models. *BMC Cancer. 18*(1), 335. doi:10.1186/s12885-018-4238-4

Wang, H., Wu, X. and Chen, Y. (2019). Stromal-Immune Score-Based Gene Signature: A Prognosis Stratification Tool in Gastric Cancer. *Front Oncol. 9*, 1212. doi:10.3389/fonc.2019.01212

Wang, X., Lang, M., Zhao, T., Feng, X., Zheng, C., Huang, C., et al. (2017). Cancer-FOXP3 directly activated CCL5 to recruit FOXP3(+)Treg cells in pancreatic ductal adenocarcinoma. *Oncogene. 36*(21), 3048-3058. doi:10.1038/onc.2016.458

Wormann, S. M., Diakopoulos, K. N., Lesina, M. and Algul, H. (2014). The immune network in pancreatic cancer development and progression. *Oncogene. 33*(23), 2956-2967. doi:10.1038/onc.2013.257

Wu, X., Sun, A., Yu, W., Hong, C. and Liu, Z. (2020). CXCL10 mediates breast cancer tamoxifen resistance and promotes estrogen-dependent and independent proliferation. *Mol Cell Endocrinol. 512*, 110866. doi:10.1016/j.mce.2020.110866

Xu, Z., Pothula, S. P., Wilson, J. S. and Apte, M. V. (2014). Pancreatic cancer and its stroma: a conspiracy theory. *World J Gastroenterol. 20*(32), 11216-11229. doi:10.3748/wjg.v20.i32.11216

Yan, Y., Gao, R., Trinh, T. L. P. and Grant, M. B. (2017). Immunodeficiency in Pancreatic Adenocarcinoma with Diabetes Revealed by Comparative Genomics. *Clin Cancer Res. 23*(20), 6363-6373. doi:10.1158/1078-0432.CCR-17-0250

Young, K., Lawlor, R. T., Ragulan, C., Patil, Y., Mafficini, A., Bersani, S., et al. (2020). Immune landscape, evolution, hypoxia-mediated viral mimicry pathways and therapeutic potential in molecular subtypes of pancreatic neuroendocrine tumours. *Gut*. doi:10.1136/gutjnl-2020-321016

**FIGURE LEGENDS**

**FIGURE 1.** Flowchart of the data analysis procedure.

**FIGURE 2.** Correlation between scores and clinico-pathological characteristics. **(A)** Correlation analysis of scores and gender. **(B)** Correlation analysis of scores and grade. All statistical analyses were carried out by Kruskal-Wallis rank-sum test **.**

**FIGURE 3.** Differentially expressed genes in immune scores and stromal scores of PAAD samples. **(A-B)** Heat-maps of top 20 differentially expressed genes between high score group and low score group based on immune score and stromal score with the threshold value of *p*<0.05 and |log fold change|>1 by Wilcoxon rank sum test, respectively.

**FIGURE 4.** GO and KEGG pathway analyses of differentially expressed gene. **(A)** Top 10 GO terms in cellular component, molecular function, and biological processes. **(B**) KEGG pathway enrichment analysis of indicated biological processes. Only both *p* value and *q* value less than 0.05 were considered significant.

**FIGURE 5.** The intersection of PPI network construction and univariate Cox regression analysis. **(A)** Top 30 differentially expressed genes sorted by quantity of nodes. **(B)** The top 16 differentially expressed genes in univariate Cox regression analysis with *p*<0.05. **(C)** CXCL10 was the only intersectional gene of PPI network construction and univariate Cox regression analysis.

**FIGURE 6.** CXCL10 is strongly correlated with prognosis in PAAD. **(A-B)** The expression of CXCL10 in normal and tumor samples from TCGA database and GTEx database. **(C)** Survival analysis for PAAD with high and low CXCL10 expression. **(D-E)** Validation of CXCL10 expression and survival of PAAD in GSE62452 dataset. **(F)** Forest plots of multivariate independent prognostic analysis.

**FIGURE 7.** Correlation between CXCL10 expression and clinico-pathological characteristics. Statistical analysis were based on Kruskal-Wallis rank-sum test.

**FIGURE 8.** GSEA analysis of CXCL10 expression in PAAD. **(A)** The enriched gene sets in C7 collection by the high CXCL10 expression cases. **(B)** The enriched gene sets in C7 collection by the low CXCL10 expression cases. Each color represented corresponding gene set. Genes on the right of X-axis had positively correlation with gene sets, which genes on the left of X-axis had nagetively correlation with gene sets. Only gene sets with NOM *p*-value <0.05 were considered as statistical significance.

**FIGURE 9.** The fractions of tumor infiltrated immune cells in PAAD and correlation analysis with CIBERSORT. **(A)** The fractions of 22 kinds of tumor infiltrated immune cells in PAAD. **(B)** Correlation with 22 kinds of tumor infiltrated immune cells. The numer and the size of circle in each tiny box on behalf of correlation value between corresponding two cells by Pearson coefficient test.

**FIGURE 10.** The correlation between the expression of CXCL10 and fractions of tumor infiltrated immune cells. **(A)** Comparisons of 22 kinds of tumor infiltrated immune cells between high and low CXCL10 expression groups. The blue violin diagram presented low CXCL10 expression group, and the yellow violin diagram on behalf of high CXCL10 expression group. Wilcoxon rank sum was used for the significance test. **(B-E)** The correlation between tumor infiltrated immune cells and the expression of CXCL10. Pearson coefficient test was used for the significance test.
